# Network Pharmacology-Based Mechanistic Investigation of Jinshui Huanxian Formula Acting on Idiopathic Pulmonary Fibrosis

**DOI:** 10.1155/2021/8634705

**Published:** 2021-07-07

**Authors:** Tiantian Liu, Pengli Xu, Shuishui Qi, Shaorui Ke, Qin Hu, Peng Zhao, Jiansheng Li

**Affiliations:** ^1^Henan Key Laboratory of Chinese Medicine for Respiratory Disease, Henan University of Chinese Medicine, Zhengzhou 450046, Henan, China; ^2^Academy of Chinese Medical Sciences, Henan University of Chinese Medicine, Zhengzhou 450046, Henan, China; ^3^Co-Construction Collaborative Innovation Center for Chinese Medicine and Respiratory Diseases by Henan & Education Ministry of P.R. China, Zhengzhou 450046, Henan, China

## Abstract

Idiopathic pulmonary fibrosis (IPF) is a chronic respiratory disease with high incidence, morbidity, and mortality rates. Jinshui Huanxian formula (JHF) is an empirical formula that targets the pathogenesis of lung-kidney qi deficiency and phlegm-blood stasis in pulmonary fibrosis (PF). The purpose of this study was to explore JHF's potential pharmacological mechanisms in IPF therapy using network intersection analysis. JHF's primary active components and corresponding target genes were predicted using various databases. Two sets of IPF disease genes were obtained from the DisGeNET and GEO databases and two sets of IPF drug targets were collected. The disease and drug target genes were analyzed. The JHF target genes that intersected with IPF's differentially expressed genes were identified to predict JHF's targets of action in IPF. The functions and pathways of predicted targets acting on IPF were analyzed using the DAVID and KEGG pathway databases. Finally, the resulting drug target mechanisms were validated in a rat model of PF. The initial analyses identified 494 active compounds and 1,304 corresponding targets for JHF. The intersection analysis revealed four common genes for the JHF targets, IPF disease, and anti-IPF drugs in the KEGG database. Furthermore, these genes were targeted by several JHF compounds. Seventy-two JHF targets were closely related to IPF, which suggests that they are therapeutically relevant. Target screening revealed that they regulate IPF through 18 pathways. The targets' molecular functions included regulation of oxidoreductase activity, kinase regulator activity, phosphotransferase activity, and transmembrane receptor protein kinase activity. In vivo experiments showed that JHF alleviated the degree of PF, including decreases in collagen deposition and epithelial-mesenchymal transition. This study systematically explored JHF's mechanisms to identify the specific target pathways involved in IPF. The generated pharmacological network, paired with in vivo validation, elucidates the potential roles and mechanisms of JHF in IPF therapy.

## 1. Introduction

Idiopathic pulmonary fibrosis (IPF) is a chronic respiratory disease, which is characterized by progressive fibrosis of lung parenchyma, resulting in function and respiratory failure. It is the most common pulmonary interstitial disease with an estimated incidence of 2.8–9.3 cases per 100,000 person per year in Europe and North America [1]. The IPF mortality rate is very high, and the median survival time is approximately 3 years [2]. IPF is a chronic and refractory disease that shows annual increase in incidence, related disability, and mortality. This seriously affects patient health and increases the social and economic disease burden. Although there are antifibrosis IPF drug therapies, there is no treatment that can change or reverse IPF fibrosis. Standard clinical treatments include anti-inflammatory drugs, immunosuppressants, antiacid therapy, and lung transplantation.

Drugs commonly used to treat IPF include glucocorticoids, azathioprine, cyclosporin, warfarin, N-acetylcysteine, and acid suppressants. However, these drugs may induce adverse events (AEs), such as myelotoxicity associated with cytotoxic drugs or diffuse alveolar hemorrhage [3]. Pirfenidone (PFD), nintedanib, and antiacid therapy are officially recommended for use by the American Thoracic Society, European Respiratory Society, Japanese Respiratory Society, and Latin American Thoracic Association clinical practice guidelines [4]. PFD may cause AEs that range from mild gastrointestinal reactions to severe drug reactions, leading to a discontinuation rate as high as 30% [5, 6]. The course and prognosis of IPF may differ between Asian and Western patients; the most common AE among Asian patients is diarrhea [7]. Antiacid therapy should be prescribed based on the patient's clinical indications. Compared to single-target drugs, multitarget drugs may be more effective due to synergistic effects or negative regulation of drug resistance [8, 9].

Traditional Chinese medicine (TCM), a natural chemical library, is a main component of medical practice. The characteristics of Chinese herbal compound include being multicomponent, multitarget, and having complex mechanisms of action. They can enhance body functions and reduce drug toxicity through the synergistic actions of their main active ingredients. Jinshui Huanxian formula (JHF) is an empirical formula for the pathogenesis of lung-kidney qi deficiency and phlegm-blood stasis in PF. JHF's monarch herbs are ginseng and *Radix Rehmanniae*. This blend nourishes yin and dissipates phlegm, promotes blood circulation and regulates qi, and primarily treats the lung-spleen deficiency syndrome and phlegm-blood stasis in the later stage of IPF. In clinical practice, JHF has shown significant improvements in clinical symptoms, inducing delayed down disease development and improving the quality of life [10].

Network pharmacology integrates system-level network analysis and pharmacology to gain insight into the complex mechanisms of herbal formulas used to treat complex diseases [11, 12]. In this context, the purpose of this study was to use network pharmacology as a basis to conduct comprehensive research exploring JHF's pharmacological mechanisms in IPF.

In order to explore JHF's pharmacological mechanisms associated with IPF, we used JHF-based network pharmacology to study the relationship among TCM, Chinese medicinal ingredients, target genes, and differentially expressed genes in IPF. First, through extensive data mining, we collected information on two groups of IPF-related disease genes, two groups of anti-IPF drugs, and their therapeutic targets. Second, we gathered information on JHF's bioactive compounds and identified candidate target genes using public databases. Potential vital targets and their related pathways involved in JHF-mediated effects were identified by analyzing the collected datasets. Then, based on IPF's differentially expressed genes, a network analysis was conducted to discover JHF's therapeutic targets that might target IPF's differentially expressed genes, their biological functions, and the important pathways involved in this response. Finally, JHF's predicted targets and functions were verified using in vivo experiments.

## 2. Materials and Methods

**Figure 1:**
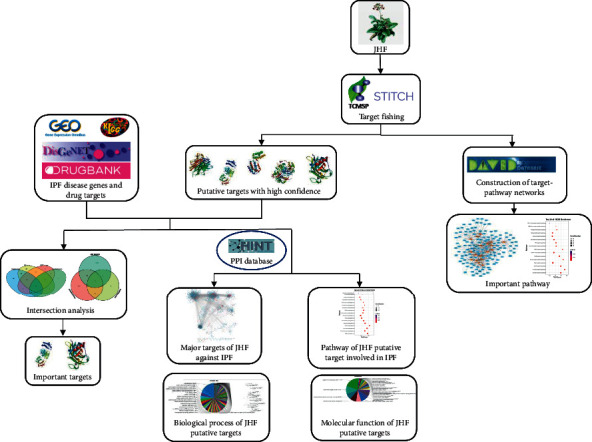
Comprehensive workflow illustrating JHF's mechanisms of treatment in IPF. The workflow includes (1) collecting JHF chemical compound information from databases and predicting their putative targets using public databases. Dataset analysis revealed the potentially important JHF targets and their relevant regulatory pathways. (2) IPF-associated genes, data for the two sets of anti-IPF drugs, and therapeutic targets were collected through extensive data mining. (3) Therapeutic targets, their biological functions, and the important pathways targeted by JHF compounds that regulate IPF genes were obtained using intersection analysis based on IPF's differentially expressed genes.

The project's workflow is illustrated in Figure 1.

### 2.1. Identification of Active JHF Ingredients

The Traditional Chinese Medicine Systems Pharmacology Database and Analysis Platform (TCMSP) (http://tcmspw.com/tcmsp.php) is a unique Chinese herbal medicine system that contains information about absorption, distribution, metabolism, and excretion (ADME) characteristics of compounds [13]. Oral bioavailability (OB) and drug-likeness (DL) are the two most important indicators to evaluate ADME characteristics using bioinformatics. OB represents the percentage of unchanged oral drug dose reaching systemic circulation, indicating the convergence of the ADME process. High OB is often the key index used to determine the properties of bioactive molecules [14]. DL is a qualitative concept used in drug design to evaluate how a “drug-like” compound responds to metrics like solubility and chemical stability, which helps optimize pharmacokinetics and drug properties [15]. The Traditional Chinese Medicines Integrated Database (TCMID) (http://119.3.41.228:8000/tcmid/search/) includes comprehensive formulas, herbs, herbal ingredients, and drug and disease information. This database helps researchers in the traditional medicine fields to discover potential new drugs and the mechanisms of drug interactions.

Using the TCMSP and TCMID, 494 active JHF ingredients were identified. Only compounds with OB ≥ 30 and DL ≥ 0.18 were retained to satisfy the criteria suggested by the TCMSP.

### 2.2. JHF Compound Targets

For each compound, putative targets were predicted from the TCMSP and STITCH (http://stitch.embl.de/, ver. 5.0) [16] using the “*Homo sapiens*” species setting. The STITCH database uses compounds that are structurally similar to JHF's chemical components to identify targets (Table S1). The threshold of confidence score was set as 0.8, a high benchmark to filter genes associated with chemicals.

### 2.3. IPF-Associated Genes

Information on IPF-associated genes was collected from the DisGeNET (http://www.disgenet.org/, ver. 6.0) [17] and GEO (https://www.ncbi.nlm.nih.gov/geo/) [18] databases. DisGeNET was searched using the disease name “idiopathic pulmonary fibrosis” to obtain 378 IPF-associated genes (Table S2). The GEO database was searched to find the genomic expression profile in lung tissues from IPF patients (GSE2052 dataset). Pardo et al. [19] ran microarray analysis on 13 IPF lung explants and 11 normal histology lung tissue samples. The screening confirmed a total of 257 differentially expressed genes with known gene symbols in IPF patients. Of these, 122 were upregulated (logFC ≥ 1) and 135 were downregulated (logFC ≤ −1) (Table S3).

### 2.4. Anti-IPF Drugs and Their Targets

Known anti-IPF drugs and their targets were collected from the KEGG [20] (https://www.genome.jp/kegg/pathway.html) and DrugBank [21] (https://www.drugbank.ca/, ver. 5.1.4) databases. Searching the KEGG pathway database revealed four IPF-associated drugs with 14 target proteins (Table S4). The DrugBank database was used to only search drugs that are approved by Food and Drug Administration for IPF treatments with human gene/protein targets. Two drugs with 26 target proteins were extracted from the DrugBank database (Table S5).

### 2.5. Construction of the Target Networks

The constructed compound-target and target-pathway networks were visualized using Cytoscape (http://www.cytoscape.org/, ver. 3.6.0).

To screen for JHF targets that may regulate differentially expressed genes in IPF, IPF-associated differentially expressed genes were first mapped to the High-quality INTeractomes database (HINT; http://hint.yulab.org, ver. 4). The HINT database is a curated compilation of high-quality protein-protein interactions from eight interactome resources (BioGRID, MINT, iRefWeb, DIP, IntAct, HPRD, MIPS, and PDB) [22]. The target gene network contained the selected targets and neighbor genes. Second, JHF's predicted targets were mapped to the IPF-associated differentially expressed gene network. A small network was extracted from the selected targets. Using this network, we obtained information regarding targets that likely regulate IPF-associated genes.

### 2.6. Functional Annotation Clustering Analysis

To clarify the functions and pathways associated with the predicted JHF targets, we used the functional annotation clustering tool in the Database for Annotation, Visualization, and Integrated Discovery (DAVID, https://david.ncifcrf.gov/home.jsp, ver. 6.8) to calculate the Gene Ontology enrichment and the KEGG pathways.

### 2.7. Reagents and Animals

JHF was provided by the Pharmaceutical Department of Henan University of Chinese Medicine. The extraction procedure used to obtain and standardize the JHF and in vivo JHF administration were performed as previously described [10]. Bleomycin hydrochloride was purchased from the Nippon Kayaku Co. Ltd. (lot 650427). PFD was obtained from the Beijing Kangdini Pharmaceutical Co. Ltd. (lot 150603; Beijing, China).

Forty Sprague Dawley rats (20 males and 20 females, 200 ± 20 g) were obtained from the Experimental Animal Center of Henan Province (Zhengzhou, China). The rats were housed under standard temperature (26 ± 2°C), humidity (50 ± 10%), and light intensity (12 h light/dark cycle) conditions and were allowed free access to standard laboratory food and water. All animal experiments were performed in accordance with international ethical guidelines and the National Institutes of Health's Guide for the Care and Use of Laboratory Animals. The experiments were approved by the Experimental Animal Care and Ethics Committee of the First Affiliated Hospital, Henan University of Traditional Chinese Medicine.

### 2.8. PF Rat Model and Drug Administration

The PF rat model was established using previously published methods [10]. The PF rats received a tracheal infusion of bleomycin at 5 mg/kg. The PF rats were intragastrically administered normal saline, JHF (10.8 g/kg), or PFD capsules (50 mg/kg) every day for 4 weeks. Finally, all rats were sacrificed and the lung tissues were collected.

### 2.9. Histological Analysis

The formaldehyde-fixed lung tissues were paraffin-embedded and cut into 4 *μ*m sections. The sections were stained with hematoxylin-eosin solution (Solarbio, Beijing, China) and Masson's Trichrome Stain Kit (Solarbio, Beijing, China) to determine the collagen distribution. Digital images were captured using light microscopy.

### 2.10. Western Blot

Lung tissues were homogenized and lysed with RIPA containing PMSF (Solarbio, Beijing, China) for 30 min and centrifuged at 12,000 g for 10 min at 4°C. Lysates were mixed with SDS loading buffer and boiled at 100°C for 5 min. The proteins were separated using SDS-PAGE gel and transferred to PVDF membranes (Millipore, Bedford, MA, United States). The membranes were sealed with 5% nonfat milk and incubated with the following primary antibodies: *α*-SMA (1 : 1000, Proteintech, China), E-cadherin (1 : 1000, Proteintech, China), N-cadherin (1 : 1000, Proteintech, China), and GAPDH (1 : 5000, Proteintech, China) at 4°C overnight. The membranes were incubated with horseradish peroxidase-linked anti-rabbit or anti-mouse antibody (1 : 3000, Proteintech, China) for 2 h and visualized using a Bio-Rad ChemiDoc^TM^ MP System (Bio-Rad, United States) with Super ECL Plus reagent (Solarbio, China).

### 2.11. Statistical Analysis

All data are expressed as mean ± standard error of the mean. Statistical analysis was performed using one-way ANOVAs followed by post hoc analysis with Tukey's tests. Results were considered significant at *P* value  < 0.05.

## 3. Results

### 3.1. JHF Compounds and Predicted Targets

JHF contained the following 12 herb materials with a total of 548 compounds: Renshen (*Ginseng Radix et Rhizoma*, 162 compounds), Maidong (*Radix Ophiopogonis*, 22 compounds), Dihuang (*Radix Rehmanniae*, 10 compounds), Gualou (*Fructus et Semen Trichosanthis*, 41 compounds), Zhebeimu (*Bulbus Fritillariae Thunbergii*, 27 compounds), Mudanpi (*Cortex Moutan Radicis*, 28 compounds), Yinyanghuo (*Herba Epimedii Brevicornus*, 50 compounds), Baiguo (*Semen Ginkgo*, 53 compounds), Baitouweng (*Radix Pulsatillae*, 25 compounds), Yiyiren (*Semen Coicis*, 11 compounds), Chenpi (*Pericarpium Citri Reticulatae*, 40 compounds), and Gouqizi (*Lycii Fructus*, 79 compounds). Fifty-four of the compounds revealed by the TCMSP and TCMID were duplicates, resulting in 494 unique compounds.

**Figure 2:**
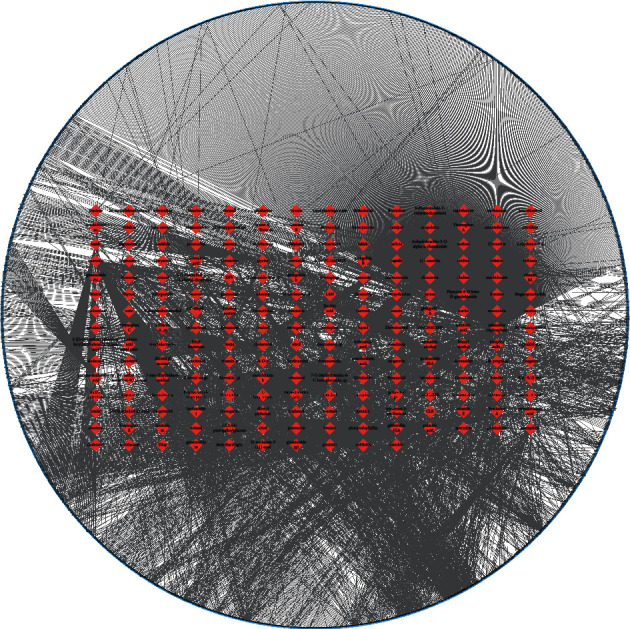
The drug-target network generated for active JHF compounds. Orange diamonds represent JHF compounds and blue nodes represent their targets.

We used the STITCH database to predict the selected compounds' targets. The compounds were predicted to interact with 1,304 distinct protein targets with a high level of confidence (Table S1).  Figure 2 shows the JHF drug-target network in a manner that describes its multicomponent and multitarget therapy. Notably, the number of mutual putative targets among the JHF compounds varied, suggesting that these herbs might have several interactions in the course of treatment.

### 3.2. Identifying JHF's Important Targets Using Intersection Analysis

**Figure 3:**
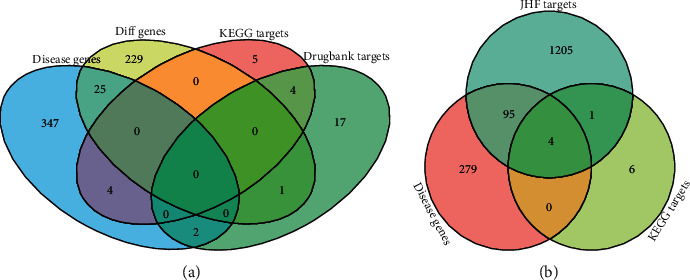
The overlap between different gene sets. (a) The overlap of disease genes from DisGeNET (Disease genes), IPF's differentially expressed genes from the GSE2052 dataset (Diff genes), and drug target genes for anti-IPF drugs in the KEGG and DrugBank database (KEGG targets and DrugBank targets). (b) The overlap between disease genes from DisGeNET, drug target genes of anti-IPF drugs from the KEGG database, and potential JHF targets.

We collected two sets of disease genes and two sets of drug targets associated with IPF for reference. We first looked at the overlap between these gene sets. Among the IPF patients who participated in the GSE2052 dataset experiment, 25 of the 378 disease genes were differentially expressed, accounting for 6.6% of all disease genes (Figure 3(a)). Notably, the KEGG and DrugBank databases revealed four drug anti-IPF drug targets, accounting for 30.8% of the target genes in the KEGG database.

Figure 3(b) shows the overlap between JHF target genes, IPF disease genes, and anti-IPF target genes in the KEGG database. Of the 1,305 JHF target genes, 99 were IPF disease genes, accounting for 7.59% of all disease genes. Among these, four were anti-IPF drug target genes: tumor necrosis factor (*TNF*), C-C motif chemokine (*CCL2*), interleukin-6 (*IL6*), and interleukin-10 (*IL10*). This suggests their important role in IPF treatment. These predictions indicated that *TNF* was targeted by 13 JHF compounds (kaempferol, quercetin, ruscogenin, luteolin, epicatechin, palmitic acid, methyl palmitate, adenosine, adenosine triphosphate, choline, ginsenoside rg1, hexadecanoic acid, and spermine), *CCL2* was targeted by six JHF compounds (naringenin, quercetin, rutin, palmitic acid, adenosine triphosphate, and hexadecanoic acid), *IL6* was targeted by eight JHF compounds (quercetin, luteolin, palmitic acid, adenosine, adenosine triphosphate, dibutyl phthalate, hexadecanoic acid, and spermine), and *IL10* was targeted by three JHF compounds (quercetin, luteolin, and adenosine) (Table S1). The four target genes were also the target of PFD, which has been approved for IPF treatment [4]. Therefore, we used PFD as a positive control in the following studies.

### 3.3. Identifying JHF-Regulated Pathways and Diseases

**Figure 4:**
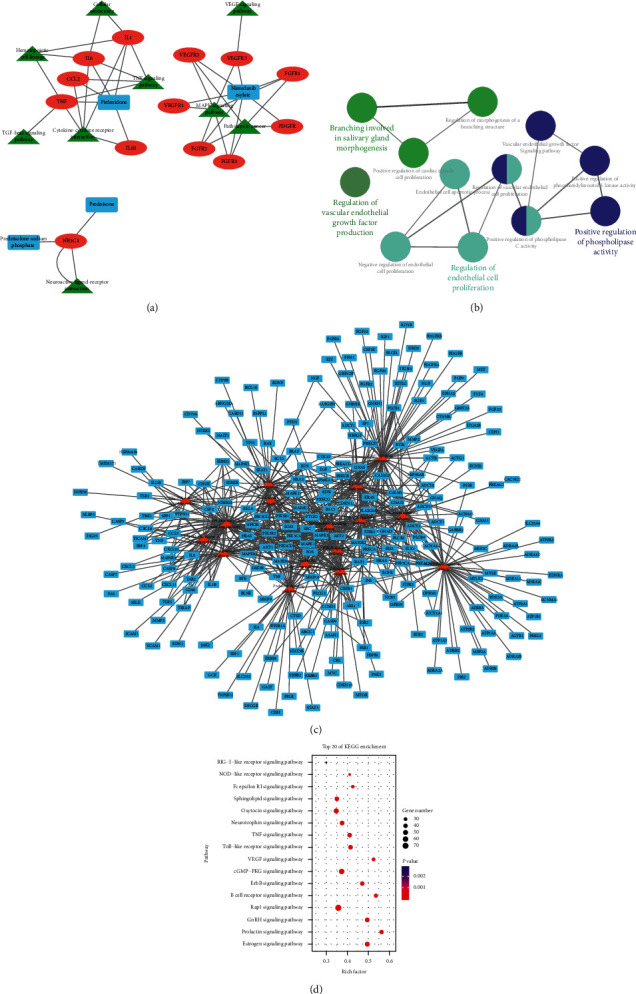
Identification of targets and pathways for anti-IPF drugs and JHF. (a) A drug-target-pathway network generated from the KEGG database search for anti-IPF drugs. Orange ellipses represent drugs, blue rectangles represent targets, and green triangles represent pathways. (b) Biological processes regulated by the anti-IPF drug targets identified in the KEGG database. (c) A target-pathway network of putative JHF targets. Blue rectangles represent targets and orange triangles represent the pathways. (d) A bubble diagram of the main enrichment pathways of putative JHF targets.

We used Cytoscape software to construct KEGG's drug-target-pathway network for the anti-IPF drugs (Figure 4(a)). PFD, a drug commonly used for PF treatment, primarily regulates processes including the TNF signaling pathway, transforming growth factor-*β* (TGF-*β*) signaling pathway, cytokine-cytokine receptor interaction, and cellular senescence. We used ClueGO, a Cytoscape plugin [23], to analyze the biological processes involved in the KEGG-identified anti-IPF drug targets. The primary biological processes identified were positive regulation of phospholipase activity, regulation of endothelial cell proliferation, branching involved in salivary gland morphogenesis and regulation of vascular endothelial growth factor (VEGF) production (Figure 4(b)).

Figure 4(c) demonstrates that target genes can participate in multiple pathways. We used DAVID analysis to elucidate the important biological pathways that JHF might regulate through its targets and constructed a target-pathway network of putative JHF targets. Because disease is an advanced biological process caused by the dysfunction of basic biological processes, we only focused on signaling pathways relevant for biological processes. JHF's targets were significantly enriched in 16 pathways ((*P* < 0.01)) (Figure 4(d)). Disease ontology enrichment was conducted to uncover the therapeutic potential of these putative targets. Through “high” strict classification and enrichment score, a total of 59 disease clusters were related to JHF targets, including inflammation, bronchiolitis, and coronary artery disease (Table S6).

### 3.4. Target, Pathway, and GO Analysis of IPF's Differentially Expressed Genes Targeted by JHF

**Figure 5:**
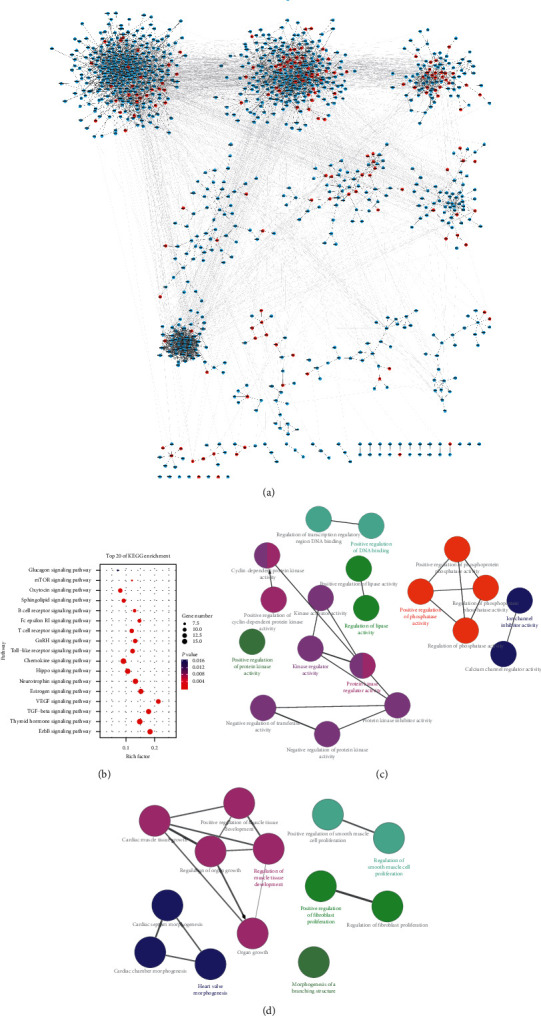
Target, pathway, and GO analysis of IPF's differentially expressed genes targeted by JHF. (a) Analysis of the disease genes differentially expressed in IPF that are regulated by JHF. Blue represents IPF's differentially expressed genes, and red represents their overlap with potential JHF targets. (b) Pathway analysis of the overlapping genes between potential JHF targets and IPF's differentially expressed genes. Molecular function analysis (c) and biological process analysis (d) of the overlapping genes.

To further improve analysis reliability, we mapped the predicted JHF targets to IPF disease gene network. This target information revealed that JHF could directly regulate differentially expressed genes in IPF (Figure 5(a)). Analyzing these targets with KEGG database revealed 72 targets, including TGF-*β*1 and SMAD3, that participated in 18 pathways, including the ErbB signaling pathway, thyroid hormone signaling pathway, and TGF-*β* signaling pathway (Table S7, Figure 5(b)). ClueGO plugin analysis through Cytoscape software revealed that the molecular functions of these targets mainly included the regulation of kinase regulator activity, protein kinase regulator activity, positive regulation of phosphatase activity, and regulation of lipase activity (Figure 5(c)). Their biological processes included the regulation of muscle tissue development, heart valve morphogenesis, positive regulation of fibroblast proliferation, and regulation of smooth muscle cell proliferation (Figure 5(d)).

### 3.5. Experimental Validation

**Figure 6:**
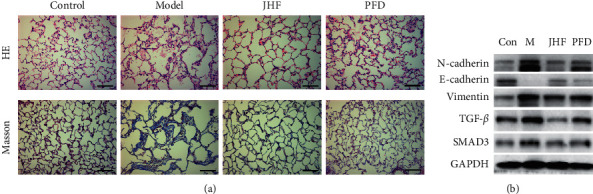
Experimental verification of relevant targets and effects. (a) Pulmonary fibrosis is ameliorated by JHF (magnification 200x). (b) Effects of JHF on epithelial-mesenchymal transition-related proteins and TGF-*β* signaling pathway proteins.

To confirm our predictions and JHF's therapeutic effects, we used a well-characterized rat model of PF that received JHF or PFD treatment. Previous studies have shown that, compared with the model group, JHF and PFD significantly attenuated decreased forced vital capacity and increased lung coefficient [10]. Our histological examinations showed structural changes in the alveoli of the PF model group, including collapsed alveolar spaces, thickening of the alveolar walls, presence of inflammatory cells, and excessive collagen fiber deposition. However, JHF and PFD alleviated this alveolar bleomycin-induced damage (Figure 6(a)). The epithelial-mesenchymal transition (EMT) contributes to the progression of fibrotic lung disease in humans [24]. TGF-*β* is an important EMT inducer and the strongest inducer of extracellular matrix deposition. Therefore, we observed EMT's role in the TGF-*β* signaling pathway during PF development. Increased vimentin and N-cadherin expression, but decreased E-cadherin expression, was observed in the PF model. This expression pattern was reversed by JHF administration (Figure 6(b)). TGF-*β* and SMAD3 expressions were also increased in the PF model group compared to the controls, but protein expression was returned to control levels by JHF administration (Figure 6(b)).

## 4. Discussion

Well-known treatment options for PF include antioxidants, cytokine inhibitors, antifibrotic drugs, and lung transplantation [25]. However, studies regarding these treatments have only focused on one or two aspects of the lung injury repair process. Although PFD has a better therapeutic effect when prescribed for PF in clinical practice, it has only received a conditional use recommendation, and its long-term efficacy and safety are not known [26, 27]. Herbal medicine has become one of the most important sources of chemical substances and lead compounds in drug discovery [28]. Network pharmacology has been used to study the complex components, unknown targets, and pharmacological mechanisms of TCM prescriptions [29]. This work presents a systematic study of the JHF's anti-IPF mechanisms from the perspective of target, pathway, network, and efficacy levels.

Identifying the IPF disease genes, JHF targets, and anti-IPF drug targets revealed four overlapping genes: *TNF*, *CCL2*, *IL6*, and *IL10*. Inappropriate TNF production is involved in the pathogenesis of many human diseases, including PF [30, 31]. Furthermore, TNF is known to affect a multitude of responses that extend far beyond its proinflammatory properties [32, 33]. CCL2 is associated with macrophage activation and may have a serious impact on the overall survival of IPF patients [34]. IL6 and IL10 play an important role in the recruitment, activation, survival, and differentiation of fibroblasts into myofibroblasts in IPF [35, 36]. Notably, although multiple JHF compounds target these genes, each compound also has other targets that differ between them. This reflects TCM's multicomponent, multitarget, and synergistic action mode characteristics.

By constructing and analyzing JHF's assumed target-pathway network that regulates IPF's differentially expressed genes, we identified five pathways that were the most significantly enriched by JHF targets: the thyroid hormone signaling pathway, chemokine, ErbB, neurotrophin, and Hippo signaling pathways.

Thyroid hormone (TH) signaling is known to play an important role in PF, including their effects on mitochondrial function. Increased transformation of prohormone thyroxine to active 3,5,3-triiodothyronine improved alveolar epithelial cell (AEC) metabolism in IPF, with thyroid hormone playing an important role in mitochondrial biogenesis and bioenergy, thereby regulating AEC apoptosis [37]. Compared to controls, IPF patients show increased activity and expression of iodothyronine deiodinase 2, an enzyme that activates TH, which was related to disease severity. TH inhibited PF through the PPARGC1A and PINK1 pathways and its antifibrosis properties were related to AEC protection and the restoration of mitochondrial function [37].

Multiple chemokines and cytokines induce fibroblast migration and induce the phenotype switch to myofibroblasts, thereby contributing to the occurrence of IPF. Chemokines and cytokines are expressed and released in alveolar macrophages, which mediate the inflammatory response and fibrotic process. During the middle stage of PF, immune cells secrete chemokines and proinflammatory factors and accelerate macrophage migration and secretion, thereby accelerating the immune response [38]. Single-cell sequencing has shown that IPF upregulates the chemokine signaling pathway [39]. Based on the RNA sequencing of bronchoalveolar lavage cells in IPF, GO identified that the chemokine-mediated signaling pathway and chemokine activity were the most significantly enriched biological processes and molecular functions, respectively. Similarly, KEGG pathway analysis indicated that cytokine-cytokine receptor interaction, the chemokine signaling pathway, and the TNF signaling pathway showed the most significant overexpression [40].

ErbB signaling is enriched in the plasma proteome of IPF patients [41]. Tyrosine kinase receptor families include Her1 (also known as epidermal growth factor receptor (EGFR)), Her2, Her3, and Her4. Several of these receptors are known to play an important role in epithelial remodeling, epithelial hyperplasia, and models of fibrosis [42–44]. EGFR plays a key role in the maintenance and repair of epithelial tissue. Identifying the interactions between c-erbB receptors and their ligands might help to determine their role in the maintenance and repair of bronchial epithelium [45].

Neurotrophin signaling is performed via the binding of NT ligands to their homologous high-affinity receptors, i.e., neurotrophic tyrosine kinase receptors. NT ligands and their homologous receptors have been detected in mice and adult human lung tissues [46–48]. Mounting evidence indicates that the expression of NTs and their homologous receptors in the NT signaling pathways is changed in lung diseases. NT3 concentrations are reduced in chronic obstructive pulmonary disease [49]. Expression of NT4/5 and its homologous receptor, TrkB, is increased in transplanted human lungs with IPF and in the lungs of mice with bleomycin-induced PF. A dysregulated TrkB/NT4/5 axis might lead to PF-related pathology, including alveolar type II cell hyperplasia and fibroblast proliferation [50].

The Hippo signaling pathway plays a key role in many important pathological processes, such as organ growth control, cell proliferation, apoptosis, tissue regeneration, and tumor suppression [51, 52]. Yes-associated protein (YAP) is a crucial downstream effector protein of Hippo. IPF patients show increased YAP activity. Interaction of the YAP and mTOR/p-S6 signaling pathways induces cell proliferation and migration while inhibiting epithelial cell differentiation [53]. Melatonin can attenuate TGF-*β*1-induced fibrogenesis in pulmonary fibroblasts by activating the Hippo pathway, resulting in the promotion of nuclear translocation and increasing YAP1 inactivation and degradation in the cytoplasm [54].

In addition, TGF-*β* is an important EMT inducer and is the strongest inducer of extracellular matrix deposition. TGF-*β* can stimulate fibroblasts to synthesize extracellular matrix components and induce matrix metalloproteinase expression [55], including SMAD-dependent and non-SMAD-dependent pathways. The SMAD complex can target many genes that activate or inhibit EMT-related transcription factor expression by interacting with DNA sequence-specific transcription factors, coactivators, or coinhibitors [56, 57]. VEGF is a potent angiogenesis promoter. Abnormal angiogenesis is a central characteristic of IPF development and progression. VEGF signaling is enriched in the plasma proteome of IPF patients, indicating its role in IPF pathogenesis [41]. In the VEGF signaling pathway, VEGF binding to VEGF receptor 2 (VEGFR2) regulates cell migration, survival, and permeability by activating PI3K in the PI3K-Akt signaling pathway [58, 59]. Some studies have shown that TGF-*β*1 can stimulate VEGF-A expression in human fetal pulmonary fibroblasts through the Smad3 signaling pathway [60]. These results indicate that targeting the VEGF and VEGF signaling pathway might produce powerful and effective IPF therapeutic targets.

Some “hub” signal molecules show multiple overlapping pathway routes that form complex functional modules [61]. This complex crosstalk among pathways and specific environmentally dependent functions allows JHF to ameliorate IPF through multiple targets and channels.

## 5. Conclusions

The current study systematically analyzed JHF's pharmacological mechanisms underlying IPF treatment to provide guidance for clinical practice. The study investigated the underlying mechanisms of a common TCM at the levels of the target, pathway, network, and biomedical efficacy; obtained information regarding JHF's therapeutic targets that might affect IPF's differentially expressed genes; and elucidated the biological functions of these targets and their important pathways. In addition, this work lays a foundation for identifying therapeutic treatments for complex diseases like IPF.

## Data Availability

The data used to support the findings of this study are included within the article and the supplementary information file.

## Supplementary Materials

Supplementary Materials Table S1 details JHF's active components and targets. Table S2 details the IPF-associated genes identified in the DisGeNET database. Table S3 describes the up- and downregulated genes in IPF patients in the GEO database. Table S4 describes the anti-IPF drugs and their targets in the KEGG database. Table S5 details the anti-IPF drugs and their targets in the DrugBank database. Table S6 is a cluster analysis of diseases related to JHF targets. Table S7 details the KEGG analysis of the targets' pathways.
